# Artificial shelters provide suitable thermal habitat for a cold-blooded animal

**DOI:** 10.1038/s41598-022-09950-y

**Published:** 2022-04-07

**Authors:** Xin Yu, Nicholas. C. Wu, Luyuan Ge, Lianshan Li, Zhengwang Zhang, Juan Lei

**Affiliations:** 1 MOE Key Laboratory for Biodiversity Sciences and Ecological Engineering, College of Life Sciences, Beijing Normal University, Beijing, 100875 China; 2 School of Life and Environmental Sciences, The University of Sydney, Sydney, NSW 2006 Australia; 3 Ecology, Evolution and Conservation, Department of Life Sciences, Imperial College London, London, SW72AZ UK; 4 Xianghai National Nature Reserve Administration, Jilin, 137215 China

**Keywords:** Behavioural ecology, Herpetology

## Abstract

Human activities such as urbanization often has negative affects wildlife. However, urbanization can also be beneficial to some animals by providing suitable microhabitats. To test the impact of urbanization on cold-blooded animals, we first conducted a snake survey at a national nature reserve (Xianghai natural reserve) and an adjacent tourist bird park (Red-crowned Crane Park). We show high presence of *Elaphe dione* in the tourist park even with high human activities and predator population (the endangered, red-crowned crane, *Grus japonensis*). We then radio-tracked 20 individuals of *E. dione*, set seven camera traps, and recorded the temperature of the snakes and artificial structures in Crane Park to document their space use, activity, and thermal preference, respectively. Our results show *E. dione* preferred to use artificial facilities to shelter from their predators and for thermoregulation. The high number of rats from the camera traps indicate abundant prey items. Overall, *E. dione* appears to be adapted to modified habitats and may expand population size at the current study site.

## Introduction

Human disturbances such as urbanization and deforestation have accelerated over the last century^1^, affecting biodiversity at a global scale^2,3^. Human activities have shown significant impact on wildlife, such as influencing their behaviours^4^, feeding schedules^5,6^, habitat use^7^, and time spent on nursing young^8,9^. Although urbanization generally has a negative effect on wildlife^10–13^, it has also been shown to provide animals living space in city habitat^14^. For example, urbanization may provide advantages for invasive amphibian populations and bolster amphibian ability to establish and spread within novel landscapes^15^. Urbanization has also benefited some insectivore bats by increasing access to insect prey and foraging habitat^16^. Therefore, it is important to evaluate the impact of urbanization and human activities on urban wildlife to help with conservation and green urban planning.

Reptiles are generally sensitive to habitat change^17^ because of their life history (low dispersal capacity and home range) and physiological dependency on environmental conditions such as temperature and water availability^18^. Therefore, reptiles are more prone to risks associated with human activities than other vertebrates^19–23^. Artificial constructions and infrastructure, a dominant landscape change in urban areas, can alter the surrounding microclimate^24–26^, which can directly affect the reptile’s ability to physiologically and behaviorally regulate^18^. For example, human recreational activities can change both nesting and basking behaviour of map turtles (*Graptemys flavimaculata*)^27^. Reptiles are commonly killed on manmade roads during mating migrations and searching for oviposition sites^28,29^. However, studies have also shown artificial constructions in suburban areas have provided suitable shelters for urban adapted lizards such as the blue-tongued lizards *Tiliqua scincoides* and their commensal prey species^30^.

Snakes are an important part of the food web and are widely distributed in various habitats, including grasslands, wetlands, forests, agriculture fields, around the residential areas, scrublands, deserts and sea^31^. As habitat destruction, environmental pollution, introduction of invasive predators, and road kills can negatively impact snake populations and health^32,33^, it is important to understand the impact of human activity and habitat modification on the activity and behavior of snakes to better inform habitat management strategies. The Xianghai National Nature Reserve of Jilin Province in China provides an interesting case study for the effects of human activity on snake abundance and activity, because a preliminary survey showed more snakes (e.g. *Elaphe dione*) were present in the Red-crowned Crane Park than in the adjacent protected area. The Red-crowned Crane Park is heavily used by tourists for bird watching, and there is high abundance of predatory birds in the park. In this study, we investigated why *E. dione*, a common snake species in the study area, prefers to live in a disturbed environment with high risk of predation. We used radio-tracking telemetry to determine space use, and camera traps to record the temperature of the snakes and artificial structures to test if snakes used the artificial structures for 1) thermoregulation, and/or 2) predator avoidance.

## Results

**Figure 1:**
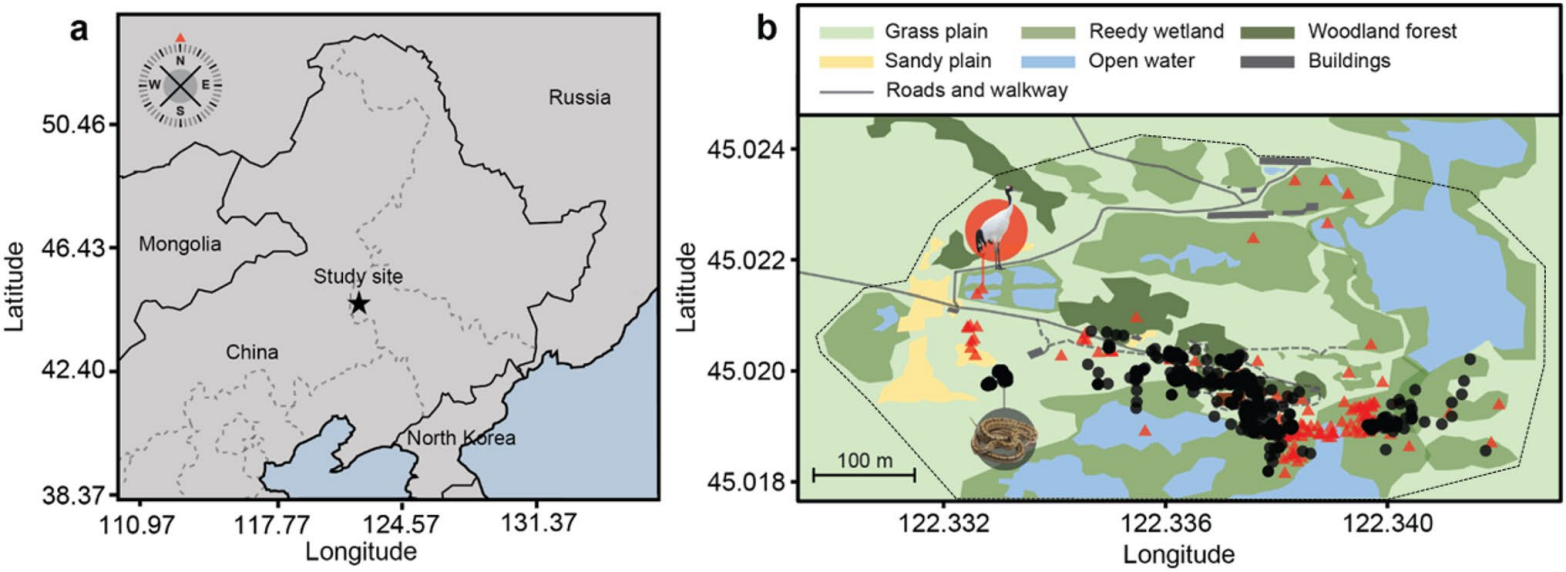
(**a**) Location of the study site at the Red-Crown Crane Park at the Xianghai Natural Reserve in Jilin province, China. (**b**) Individual GPS points of all individual *Elaphe dione* radio tracked (black circles) and their avian predator, *Grus japonensis* sightings (red triangles) during the summer period (May–July). Black dotted line around crane park presented the boundary between Red-Crown Crane Park and the Xianghai Natural Reserve. This figure was generated by using R (R × 64 3.1.3)^70^.

Of the twenty *E. dione* with radio transmitters, only 16 individuals had sufficient GPS fixes (> 30 fixed points) for further analysis. Four snakes lost radio signal during the sampling period and thus were excluded from the study. Of the 16 animals that were radio tracked (6 males, 10 females), females had higher mean body mass than males (mean ± S.D.; female = 204.8 ± 15.3 g; male = 134.2 ± 11.8 g). Individuals were tracked between 32 and 69 d (mean ± S.D.; 50.1 ± 2.8 d) (Fig. S3), with 38 to 88 fixes (mean = 70.1 ± 3.7 fixes). The space activity used during the survey period was small, about 3.1 ± 1.6 ha (0.031 ± 0.016 km^2^; Table S2). On average, male *E. dione* used larger space than female *E. dione*, however there was high variability between individuals (estimate ± 95% CI, 0.94[− 1.2–3.09]). Accounting for sex, there was a weak positive relationship between body mass (1.38[− 2.06–4.77]) and body length (3.47[− 4.81–11.8]) on space use (Fig. 1).

**Figure 2:**
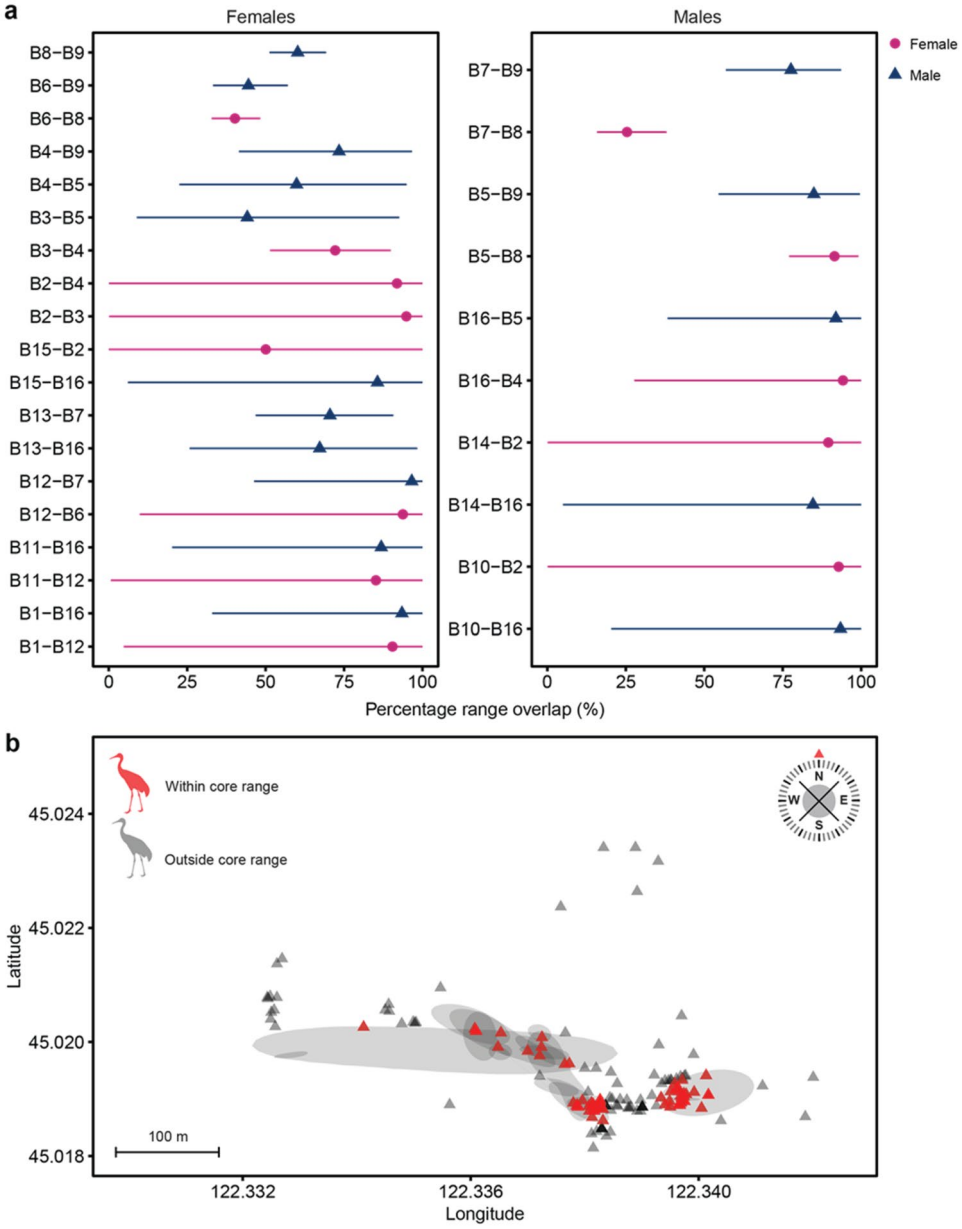
(**a**) The top two pairwise overlap estimates (± 95% confidence intervals) based on the Bhattacharyya coefficient for each *Elaphe dione* (left plot females, right plot males). Purple circles represent an individual paired with a female, while blue triangle represent an individual paired with a male. Results for all possible pairs of individuals in Table S3. (**b**) The degree of *Grus japonensis* sightings overlapping with the E. dione 50% active range (filled contours). Red riangles represent *G. japonensis* sightings in the core activity space, while black triangles represent *Grus japonensis* outside the activity space.

There was substantial overlap in space use (120 overlapping combinations), where all individuals overlapped in activity range with another individual at least once (Table S3). From the survey period, intra-sex overlap was most common (60 overlapping combinations), with moderate female-to-female overlaps (45 overlapping combinations), and the fewest with male-to-male overlap at 15 overlapping combinations (Fig. 2a). Because of the disproportionate number of females tracked in the study, the lower male-male interactions may be due to lower sample size of males. Of the 158 crane points observed during the survey period, 86 points (54.4%) were located within the *E. dione* 50% core activity range (Fig. 2b).

### Snake abundance survey

Across the five survey days at each location, we did not observe any snake species at the Xianghai National Nature Reserve, while nine individuals of *E. dione* were observed in Red-crowned Crane Park.

### Habitat selection

Manmade area (*Bi* = 0.919) was preferentially used above other habitat types. The grassland, reedy wetland, sandy plain (*Bi* = 0.031, 0.020, and 0.028, respectively) were occasionally used, and woodland forest was rarely occupied (*Bi* = 0.003; Table 1). *Elaphe dione* never occupied open water areas (Table 1).

**Table 1:** Calculation of standardized habitat index (*B*_*i*_) as a proportion of selection index (Ŵ) based on radio tracking for *E. dione*.

Habitat	Population proportion (π_i_)	Sample count	Sample proportion (*O*_*i*_)	Selection index (Ŵ)	Standardized habitat index (*B*_*i*_)
Grassland	41.08	417	37.30	0.91	0.031
Reedy wetland	29.11	191	17.08	0.59	0.020
Open water area	19.20	0	0	0	0
Woodland forest	5.32	5	0.45	0.08	0.003
Sandy plain	3.75	35	3.14	0.83	0.028
Manmade area	1.54	470	42.04	27.30	0.919
Total	100	1118	100	29.71	1.00

Population proportion (π_i_) is calculated as a percentage of the total area, for each of the habitats covered. Sample counts are the number of fixes in each habitat. Sample proportion (*O*_*i*_) is the sample count as a percentage; selection index is calculated by Ŵ = *O*_*i*_/π_i_.

### Body and environmental temperature

**Figure 3:**
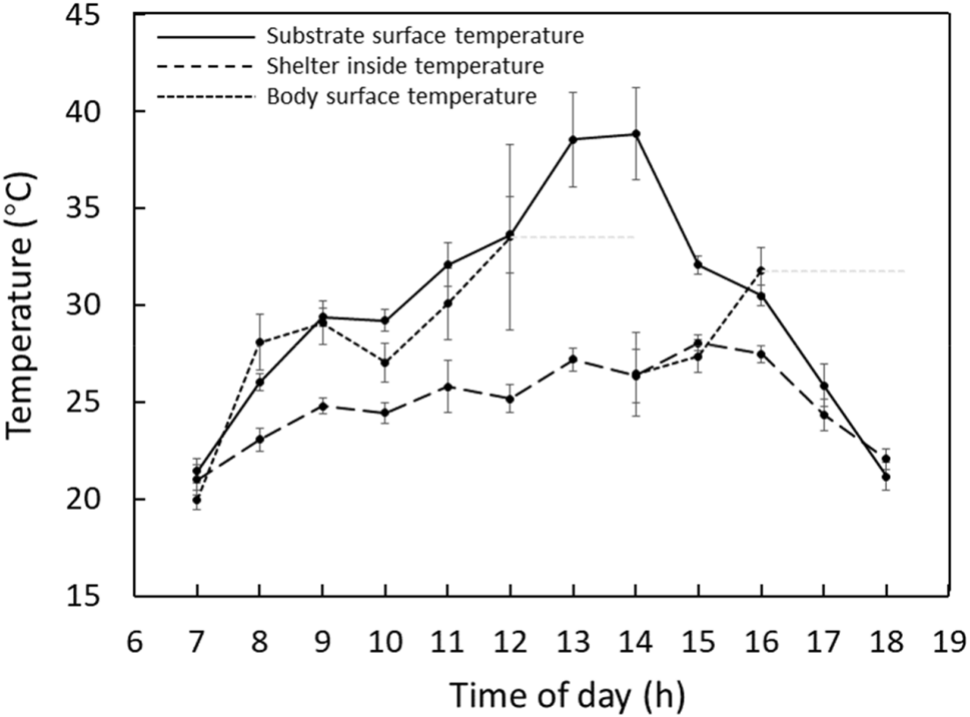
Relationship between substrate surface temperature (°C, solid line), shelter inside temperature (°C, long dotted line) and body surface temperature (^◦^C, short dotted line) from 16 *E. dione* during study period (1st May–25th July 2021). Light grey short dotted line represents no data collected during the period. Mean ± S.E. for each hour of the day is shown.

Temperature data recorded during our radio tracking period showed daily substrate temperature patterns followed a unimodal pattern, with temperature rising steeply to reach a peak of 35–40 °C between 07:00 and 13:00 h, and then falling steeply between 15:00 and 18:00 h and dropping to 20 °C at 18:00 (Fig. 3). Daily shelter inside variation in temperature was small, with temperature gently fluctuating between 21 °C and 27 °C. Substrate surface temperature was significantly higher than that inside of the shelter during midday where substrate temperature and shelter inside temperature were 33.5 ± 1.1 °C and 25.5 ± 0.6 °C (P < 0.001), respectively. Tracked snakes’ body surface temperature presented two rising periods, 13:00 to 12:00 h and 14:00 to 16:00 h. None of snakes were sighted between 12:00 to 14:00 h and 17:00 to 18:00 h respectively during tracking, and thus no body surface temperature was recorded during these periods.

### Camera traps

Camera traps showed three snake species, *E. dione*, *Orientocoluber spinalis* and *Rhabdophis tigrinus*, that visited monitored sites during the study period (Fig. S2a,b,c). Rodents (38 events) were the most common animals captured by camera traps (Fig. S2d). All monitored sites had at least three images of snakes visit during the deployment period, with 97 visitation events from snakes recorded; 80 (82.5%) of these visitation events were made by *E. dione*, nine (9.3%) were made by *O. spinalis*, and eight (8.2%) were made by *R. tigrinu*.

**Figure 4:**
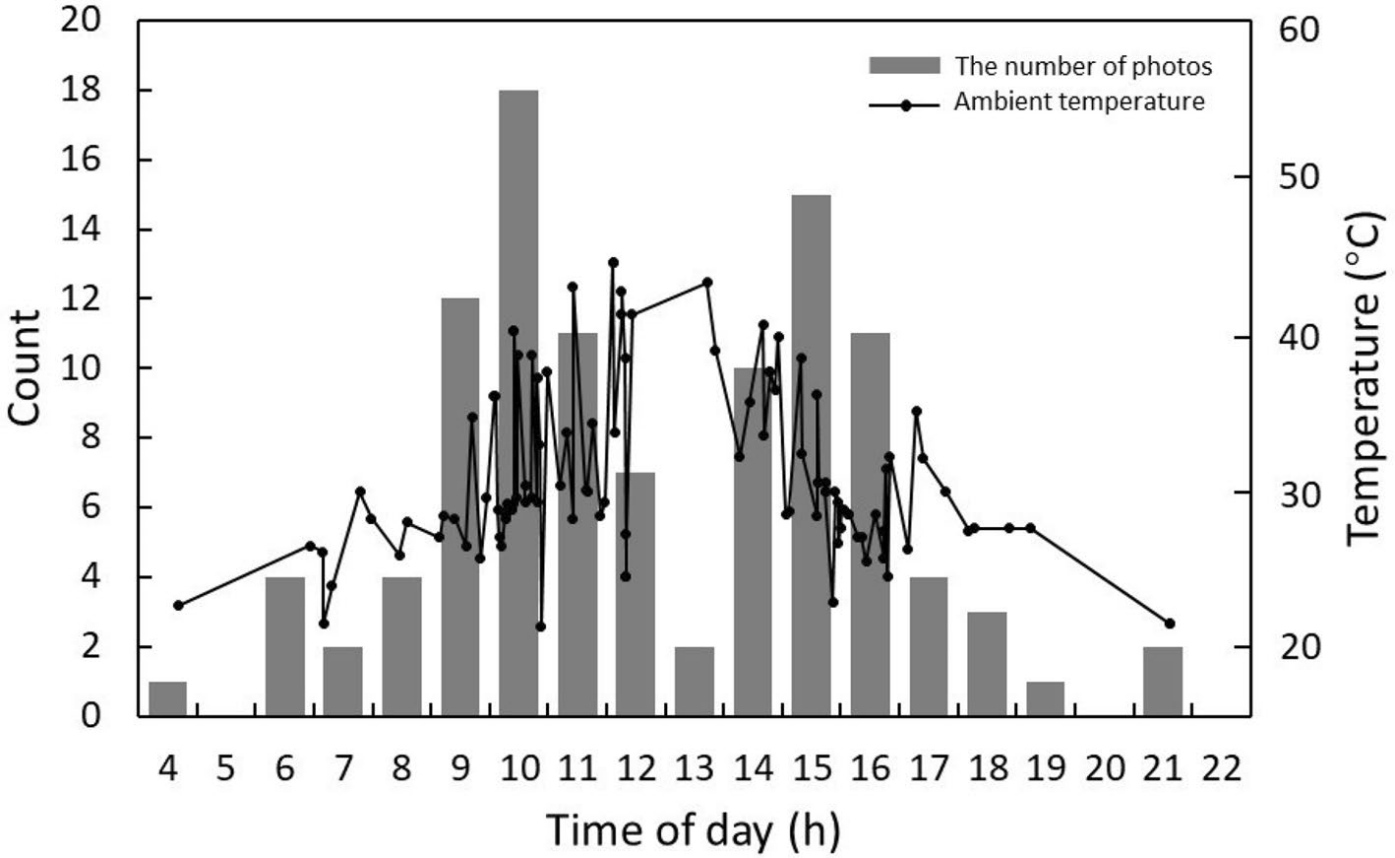
Plot of the number of *E. dione* images taken with the camera traps across the day. The number of images of *E. dione* taken by camera traps set at study area in relation to time of day and ambient temperature that images were recorded.

*Elaphe dione*, *O. spinalis* and *R. tigrinus* were active throughout the sampled time points between 4:00–21:00, 11:00–17:00 and 07:00–16:00. However, all three species showed a strong bi-modal pattern of activity, with a peak in activity in the morning between 9:00 and 11:00, and again in the afternoon between 14:00 and 16:00 (Fig. 4). The air temperature followed a unimodal pattern, with temperature rising to a peak of 45 °C from 07:00 to 12:00 h, and then falling steeply to 20 °C between 12:00 and 17:00 h (Fig. 4).

## Discussion

Our results indicate that *E. dione* has a strong preference for the artificial area with a relatively small activity range indicating high site fidelity. During our tracking period, we observed *E. dione* preferred to bask around their shelters such as concrete crack and space between bricks (Fig. S1b). When *E. dione* noticed a predator approaching, they rapidly move in these hiding sites, which may explain why *E. dione* live in an area with high crane population. We expected snakes may behave differently in the reserve site because they may rely on rodent holes or tree cracks. Future studies are required to compare snakes’ behaviours and site choices between natural and artificial conditions. Shelters provided protection against predators and buffer environmental conditions, which can be especially true for non-burrowing ectotherms, including most snake species, which rely on existing shelters^34,35^. The habitat at the Xianghai National Nature Reserve is mainly grassland with limit shelters for snakes relative to the Red-Crowned Crane Park. This may be the reason why the snake abundance is high at the high human active park in this study. However, due to the short survey efforts (5 days), our results may not accurately capture snake abundance in the Xianghai National Nature Reserve given the large reserve size. Nevertheless, there was high abundance observed in Red-Crowned Crane Park within a given area of survey.

Our radio tracking showed that both male–male and male–female *E. dione* home ranges overlap to a large degree, indicating individuals of this species do not exclude conspecifics from their home ranges. In addition, the three snake species (Fig. S2) observed shared a same shelter by our camera traps, implying tolerance to intraspecifics. Detailed examination of date and time records of GPS locations of individuals showed that vast individuals were found at same hiding area (98 encounters in 75 days of tracking). Such site-fidelic behaviour can be found in garter snakes *Thamnophis sirtalis sirtalis*^36^, tiger snakes *Notechis scutatus*^37^ and *Boa constrictor*^38^. Mutual attraction and aggregation may serve multiple functions such as mating, food finding, and group vigilance against predators^39,40^. However, more field observations are required to test these potential aggregation functions in *E. dione*. It is possible that *E. dione* use their excellent sense of smell to detect and remember where the aggregation area is^41^.

Given the exposed nature of the open artificial habitat, which results in direct exposure solar radiation, we expected these snakes would use their artificial shelter (e.g. concrete crack) for thermoregulation from the extreme heat during the middle of the day as reported in other reptiles that live in hot habitats^42–45^. Similar to these expectations, we did not observe that *E. dione* outside of their shelters during the hottest part of the day (12:00 to 14:00 h) from our radio tracking and camera traps. Hiding inside of the concrete or brick shelters resulted in shelter temperatures between 24–27 °C during mid-day, which were 9–18 °C lower than the surrounding substrate surface temperature (33–45 °C). The hottest part of the day often exceeds the physiological thermal limits of most terrestrial ectotherms^42,46^, and many ectotherms, including snakes, behaviorally thermoregulate by seeking cooler parts of their microhabitats to maintain optimal body temperatures or avoid reaching their upper thermal tolerance limit^47^. We therefore interpret *E. dione* sheltering in cooler environments during the hottest period of a day. In the context of climate warming, such artificial structures may play an important in behaviorally adapting to climate extremes such as heat waves. Based on the current study, *E. dione* always retreated to their shelter in the presence of cranes, and thus cranes may not easily capture them even though their activity area overlapped. Snakes often use other animal burrows to shelter from predators^48^. Thus, conservation strategies are often designed by providing artificial refugia. Zappalorti and Reinert^49^ constructed subterranean refugia and successful attracted corn snakes *Elaphe guttata* and pine snake *Pituophis melanpleucus* for shelter, ovipositing, courtship, shedding and basking. For the threatened lizard, *Timon lepidus*, the artificial refuges were rapidly occupied by lizards, suggesting that this technique was successful to improve lizard habitat^30^. Therefore, further conservation strategies for endangered snake species are encouraged to provide artificial shelters to against predation and microhabitat loss.

In addition to predator avoidance and thermoregulation, our data from camera traps also show these manmade constructions supported a large number of rodents residing in the park which may provide additional prey items for *E. dione*. The presence of human waste and crane food leftovers may attract rodents or small sized bird species foraging in this area, result in increasing the prey abundance for *E. dione*. Traditional snake conservation strategies include translocations, reintroductions, habitat manipulations and the creation of artificial hibernation sites^50,51^, whereas, studies testing the effectiveness of these management strategies are generally limited. Our study highlights the inclusion of predator–prey information and thermal data for conservation strategies^52^. Future studies are required to examine appropriate artificial shelter could promote endangered snake species’ survival.

Our study provided important insights into the spatial use and behavior in predator avoidance and thermoregulation of *E. dione*. First, *E. dione* preferred to use artificial habitat as shelter in the presence of high predator (red-crowned crane) population. Second, *E. dione* used their shelter to avoid high ambient temperatures during the middle of the day and were not active during this period. The manmade constructions experienced relatively constant internal temperatures during the middle of the day, and the lack of predation suggests that *E. dione* are able to persist in urbanized areas with high human activity and predators.

## Methods

### Ethics statement

All procedures were designed to minimize stress to the animals, were carried out in accordance with relevant guidelines and regulations (including the Animal Research: Reporting of In Vivo Experiments (ARRIVE) guidelines), and all experimental protocols were approved by the Beijing Normal University animal care and ethics committee (Approval no. CLS-EAW-2021–034).

### Study area

This study was conducted at the Xianghai National Nature Reserve of Jilin Province (Fig. 1a), which is located on the Northeast of mainland China (45.0158–45.1390 N, 122.2568–122.3900 W; GPS datum ¼ WGS84; Fig. 1a) which covers 20,218.8 ha and the adjacent Red-crowned Crane Park (Fig. 1b) which occupies 84.1 ha. Xianghai has a northern temperate climate with peak temperature during the summer (May–August) averaged 23.4 °C. The study site supports an abundant population of *Elaphe dione*^53^, although population estimates have not been quantified . Both Xianghai National Nature Reserve and Red-Crowned Crane Park are mostly covered in grassland, reedy wetland and lakes. Xianghai National Nature Reserve is not open for public, whereas the Red-crowned Crane Park has several buildings constructed for keeping and breeding red-crowned cranes, and artificial ground and roads for tourist (Fig. 1b). The Red-crowned Crane Park contains approximately 200 captive-bred red-crowned cranes *Grus japonensis* and around 40 of them are free-ranging with providing limited food. During tourist season (from May to November), about 100–500 tourists per day visited the park.

### Study species

*Elaphe dione*, is a diurnal Colubridae snake that widely distributed throughout Western Europe, Russia and East Asia, including China and Korea^53^. This species is found in a diverse range of habitats, including forests, wetland, grassland agricultural lands, and mountainous region^53,54^. Observations studies show this species regularly feeds on small mammals, birds, lizards and frogs^53^. Carnivorous mammals, birds and ophiophagous snakes (e.g. *Dolichophis caspius*) are known predators of *E. dione*^53,55^*.*

### Snake abundance survey

In early May, we surveyed the snake population abundance at the Xianghai National Nature Reserve and Red-crowned Crane Park. Each location was surveyed for five continuous days by walking patrolling with a scale of 15 km at Xianghai National Nature Reserve and 3 km at Red-crowned Crane Park respectively. Since the Xianghai National Nature Reserve is large, five people were allocated to this location, whereas two people were allocated to Red-Crowned Crane Park. Once a snake was observed, they were identified, and their locations were recorded.

### Animal capture, tagging, and release

During summer, 2021 (1 May–23 July), 20 adult *E. dione* only in Red-Crowned Crane Park were captured by hand. All snakes captured did not have obvious food items in their stomach. Once caught, the snout-to-vent length (SVL) and total length were measured with a flexible fiberglass measuring tape (± 0.1 cm), mass was recorded by a digital scale (± 0.1 g), and the sex determined by squeezing the tail base to evert the hemipenis of males or examining the thickness and scales at the tail base^56^. If hemipenes could not be everted, we inserted a blunt probe into the cloaca and probed in a posterior direction towards the tip of the tail to confirm sex. Probe depth of males is generally longer than that of females in reptiles^57^. A radio transmitter (Model BD-2, HOLOHIL Canada, 1.6 g, which was, 0.9% of average *E. dione* body mass; average mass of *E. dione* with transmitter 180.0 ± 13.6 g) was then attached to the dorsal surface of the base of the tail using subdermal stitch (Fig. S1a) following Riley et al^58^. The transmitter frequencies were set within the 150–151 and 216–217 MHz band. All snakes with implanted transmitters were held briefly in a temporary housing at Crane park to recover from the implanted transmitter and then released at their site of capture within 24 h of processing. The transmitters are manually removed when the tracking duration was completed. Each snake was tracked for 60 d at three time periods per day during the active period with focus on the morning (07:00 to 11:00 h), midday (11:01 to 14:30 h), and afternoon (14:31 to 18:30 h). Each individual location was found by using the increase in transmitter signal strength received by the receiver (Model R1000, Communications Specialists, inc, Japan) as the snake was approached and its fixed position recorded using a handheld global positioning system (GPS; Garmin eTrex 30; Estimated Position Error [EPE] = 5 ± 1.2 m) once confirmed by visual sighting. In addition, red-crowned cranes were recorded by patrolling the park area with focus during active periods^59^: the morning (06:00 to 11:00 h), midday (11:01 to 14:30 h), and afternoon (14:31 to 18:00 h). Then a crane was located its position (fix) was recorded using a handheld global positioning system (GPS; Garmin eTrex 30; Estimated Position Error [EPE] 5 ± 1.2 m).

### Temperature measuring

Due body surface/scale temperature of a snake is strongly correlated with its body temperature^60^, each time the tracked snake was sighted, the body surface temperature of snakes and the substrate surface temperature immediately adjacent to the snake (within a radius of 3–5 cm) were measured with infrared thermometers (BD380, Brady, China accurate to ± 1.0 °C). If located snake was hiding under a shelter, both the outside temperature was measured by infrared thermometers (BD380, Brady, China; accurate to ± 1.0 °C), and the temperature 10–15 cm inside of shelter was measured with a thermocouple thermometer (Omega 871A type K; accurate to ± 0.5 °C).

### Camera traps

Camera traps were used to monitor activity levels of snakes and their prey around known aggregation sites to test for activity correlations with the ambient air temperature and predator avoidance if a predator appears. Seven camera traps (LTL-6210 Plus series, LTL ACORN, UK) with inbuilt temperature sensors were set up to capture images of snakes adjacent to a sample of seven snake aggregation sites (e.g. concrete crack, rat holes and building roof) at crane park between 21 May 2021 and 24 July 2021. Camera traps were set at each location for 30 days. All camera traps were triggered by motion sensors and could be triggered 24 h per day. The triggers were set as sensitive to be able to detect slow moving snakes. Camera traps were positioned 30 cm face the selected location. Each camera trap had a 0.5 m^2^ field of view over the nest ensuring that any snake activity was recorded. This enabled information on the frequency, time of day, ambient temperature and species to be collected.

### Data analysis

#### Space use estimation

Space use was defined as an area used by an animal throughout the study duration^61^. We calculated variograms, fit movement models, and estimated spatial probability density functions via the *ctmm* package^62^ for each individual via an area-corrected, autocorrelated kernel density estimation (AKDE_C_) that was optimally weighted to determine the distribution of occupancy using the continuous-time movement model. The AKDE_C_ fits the telemetry data a continuous time stochastic process movement model to fit an appropriate kernel bandwidth to account for spatiotemporal autocorrelation, particularly at small sample sizes^63^. Space use and movement parameters for each individual was estimated visually via variograms^64^ and an automated model selection via the perturbative hybrid residual maximal likelihood (pHREML^65^) which considers a suite of potential movement models with either an anisotropic (asymmetrical diffusion) and isotropic (symmetrical diffusion) version of the Ornstein–Uhlenbeck motion model (UO^66^), the Ornstein–Uhlenbeck motion model with foraging-process (OUF; Gaussian estimates of the root mean speed^64^), a special case of the OUF model (OUf) where the velocity autocorrelation timescale and the position autocorrelation timescale are equal, and the independent and identically distributed model (IID). Model selection was based on Akaike’s Information Criterion adjusted for small sample sizes (AIC_C_; Table S2). For individuals with high pHREML estimate bias (1 /*N*^2^ > 2%), where *N* is the effective sample size from the initial pHREML estimate (Table S2), we used parametric bootstrapping pHREML (bpHREML) to reduce effective sample size bias in the model estimates^65^. We estimated the 95% space use and the area encompassed by the 50% AKDE_C_ isopleth, the proportion of the total (95%) space use area contained by the 50% core activity (Fig. S4).

#### Biometric correlates of space use

We tested if body size (SVL and mass) or sex predicted the variation in space use by constructing Bayesian regression models via the ‘brms’ function from the *brms* package^67^. We constructed four chains of 5,000 iterations per chain, including 2,000-step warm-up periods so a total of 10,000 steps were retained to estimate posterior distributions (i.e. (5,000 – 2,500) × 4 = 10,000). Adapt delta was set at 0.99 to decrease the number of divergent transitions, and the max tree depth was set to 15 when the depth of tree evaluated in each iteration was exceeded. We conducted model fitting with either the natural logarithm-transformed SVL or body mass with sex as a covariate. All models were assigned default priors, and the degree of convergence of the model was deemed as achieved when the Gelman–Rubin statistics, $\widehat{R}$ (Gelman & Rubin, 1992) was 1. Results were presented as mean Bayesian estimates ± 95% credible intervals.

#### Individual space use overlap

Range overlap was quantified using the Bhattacharyya coefficient^68,69^ on the 95% AKDE_C_ estimates via the ‘overlap’ function in the *ctmm* package. The Bhattacharyya coefficient calculates the relative overlap between two probability distributions, and therefore allows calculation of uncertainty in the AKDE_C_ estimates with a range between 0 (no overlap) and 1 (complete overlap). Range overlap was presented as percentage overlap ± 95% confidence interval.

#### Temperature profile

Differences between the temperature profile of the substrate surface, shelter outside and inside temperature across the day were compared with an analysis of variance (ANOVA) in R (R × 64 3.1.3)^70^. Comparisons were reported as mean ± S.E. and *P* < 0.05 was considered to be significant.

#### Habitat preferences

To determine if *E. dione* had a preference for a particular habitat type, fixes were first plotted on landscape maps by combined visual mapping of habitat type with data imported from Google maps using the adehabitatHR package^71^ in *R*, so that each fix could be assigned to a habitat type. A habitat selection index (Ŵ) was calculated for each of the habitat types (sandy plain, bushland, rainforest, mangrove and walkway) using the Eq. ^72^:

$${\hat{\text{W}}} = O_{i} /\pi_{i}$$

where *O*_*i*_ = the proportion of the population sampled in each habitat type, and π_*i*_ = the proportion of total study area each habitat type covers. Then the standardized habitat selection index (*B*_*i*_) for each habitat type was calculated using the Eq. ^72^:

$$B_{i} = {\hat{\text{W}}}_{i} /\sum {{\hat{\text{W}}}}$$

## Supplementary Information

Supplementary Information.

## Data Availability

All datasets generated and analysed are available on the Github repository: https://github.com/Juan881204/Artificial-shelters-provide-suitable-thermal-habitat-for-a-cold-blooded-animal.
