# Supplementary material for: Artificial shelters provide suitable thermal habitat for a cold-blooded animal
Source: Sci Rep. 2022 Apr 7;12:5879. doi: 10.1038/s41598-022-09950-y (PMC8991271; doi:10.1038/s41598-022-09950-y)
Supplement: Supplementary file 1 — Supplementary Information. [file 41598_2022_9950_MOESM1_ESM.docx]

**SUPPLEMENTARY FILE**

**Supplementary Tables**

**Table S1.** Biometric description of the sex (F = female, M = male), body size, snout-vent-length, as well as the total days of tracking and the number of GPS fixes of 16 adult *Elaphe dione*.

| **Animal ID** | **Sex** | **Body mass (g)** | **Snout-vent-length (cm)** | **Days tracked** | **GPS fixes** |
| --- | --- | --- | --- | --- | --- |
| B1 | F | 302.86 | 93.5 | 69 | 88 |
| B2 | F | 233.3 | 82.5 | 34 | 59 |
| B3 | F | 191.45 | 76.8 | 53 | 80 |
| B4 | F | 120.96 | 75.4 | 52 | 76 |
| B5 | M | 151.03 | 73 | 44 | 73 |
| B6 | F | 233.16 | 92.4 | 50 | 84 |
| B7 | M | 111.64 | 64.7 | 63 | 88 |
| B8 | F | 199.75 | 88.5 | 44 | 62 |
| B9 | M | 122.34 | 77 | 59 | 78 |
| B10 | M | 153.72 | 80.8 | 59 | 79 |
| B11 | F | 219.5 | 87.5 | 48 | 65 |
| B12 | F | 197.81 | 85.3 | 32 | 38 |
| B13 | F | 156.44 | 81.5 | 62 | 84 |
| B14 | M | 95.3 | 74 | 38 | 62 |
| B15 | F | 193.2 | 83.2 | 36 | 56 |
| B16 | M | 171.4 | 89.8 | 58 | 49 |
| Mean ± s.e | . | 178.4 ± 13.6 | 81.6 ± 2.0 | 50.1 ± 2.8 | 70.1 ± 3.7 |

**Table S2.** Area-corrected, autocorrelated kernel density estimation of area of occupancy estimation ± 95% CI (km²), the effective sample size (*N*), observed sample size (*n*), expected bias (1/*N*²), expected bias – bootstrapped (1/*N*³), and whether the samples were bootstrapped prior to space use estimation. Movement models were chosen based on the Akaike’s Information Criterion adjusted for small sample sizes via the perturbative hybrid residual maximal likelihood.

| **Animal ID** | **Movement model** | **Area (km²)** | **2.5% CI** | **97.5 % CI** | ***N*** | ***n*** | **Expected bias (%)** | **Expected bias - Bootstrapping (%)** | **Bootstrapped** |
| --- | --- | --- | --- | --- | --- | --- | --- | --- | --- |
| B1 | OU anisotropic | 0.026 | 0.009 | 0.053 | 5.02 | 88 | 3.97 | 0.6 | yes |
| B10 | IID anisotropic | 0.007 | 0.005 | 0.009 | 6.86 | 79 | 2.12 | 0.0005 | yes |
| B11 | OU anisotropic | 0.002 | 0.001 | 0.003 | 5.79 | 65 | 2.98 | 0.01 | yes |
| B12 | OU anisotropic | 0.025 | 0.016 | 0.037 | 23.48 | 38 | 0.1 |  |  |
| B13 | OU isotropic | 0.005 | 0.003 | 0.007 | 16.92 | 84 | 0.3 |  |  |
| B14 | OU anisotropic | 0.001 | 0.001 | 0.001 | 0.69 | 62 | 209.7 | 0.0004 | yes |
| B15 | IID anisotropic | 0.056 | 0.033 | 0.084 | 18.39 | 56 | 0.2 |  |  |
| B16 | OU anisotropic | 0.274 | 0.163 | 0.413 | 1.61 | 48 | 38.7 | 0.016 | yes |
| B2 | IID anisotropic | 0.002 | 0.001 | 0.002 | 0.02 | 59 | 32858 | 0.05 | yes |
| B3 | IID anisotropic | 0.007 | 0.005 | 0.009 | 49.05 | 78 | 0.01 |  |  |
| B4 | OU anisotropic | 0.016 | 0.008 | 0.028 | 9.54 | 75 | 1.09 |  |  |
| B5 | OU anisotropic | 0.014 | 0.006 | 0.027 | 3.87 | 73 | 6.67 | 0.3 | yes |
| B6 | IID anisotropic | 0.007 | 0.005 | 0.008 | 81.81 | 84 | 0.01 |  |  |
| B7 | OUF anisotropic | 0.016 | 0.007 | 0.029 | 8.40 | 88 | 1.4 |  |  |
| B8 | OU anisotropic | 0.009 | 0.007 | 0.012 | 43.98 | 62 | 0.05 |  |  |
| B9 | OU anisotropic | 0.031 | 0.020 | 0.045 | 23.48 | 78 | 0.1 |  |  |

**Table S3.** Bhattacharyya coefficient probability of range overlap [estimate ± 95% confidence interval (CI)] for all pair combinations.

| **Animal ID** | **Pairwise** | **2.5% CI** | **Estimate** | **97.5% CI** |  | **Animal ID** | **Pairwise** | **2.5% CI** | **Estimate** | **97.5% CI** |
| --- | --- | --- | --- | --- | --- | --- | --- | --- | --- | --- |
| B1 | B10 | 0.33 | 0.73 | 0.99 |  | B14 | B5 | 0.05 | 0.33 | 0.86 |
| B1 | B11 | 0 | 0 | 0 |  | B15 | B5 | 0.01 | 0.14 | 0.66 |
| B10 | B11 | 0.00 | 0.14 | 1.00 |  | B16 | B5 | 0.38 | 0.92 | 1 |
| B1 | B12 | 0.05 | 0.90 | 1 |  | B2 | B5 | 0.0004 | 0.88 | 1 |
| B10 | B12 | 0.15 | 0.89 | 1 |  | B3 | B5 | 0.09 | 0.44 | 0.93 |
| B11 | B12 | 0.01 | 0.85 | 1 |  | B4 | B5 | 0.23 | 0.60 | 0.95 |
| B1 | B13 | 0.05 | 0.31 | 0.83 |  | B1 | B6 | 0.28 | 0.67 | 0.97 |
| B10 | B13 | 0.001 | 0.04 | 0.45 |  | B10 | B6 | 0.00 | 0.17 | 0.93 |
| B11 | B13 | <0.0001 | <0.0001 | <0.0001 |  | B11 | B6 | 0 | 0 | 0 |
| B12 | B13 | 0.31 | 0.93 | 1 |  | B12 | B6 | 0.10 | 0.94 | 1 |
| B1 | B14 | 0.07 | 0.32 | 0.79 |  | B13 | B6 | 0.09 | 0.18 | 0.33 |
| B10 | B14 | 0.06 | 0.35 | 0.85 |  | B14 | B6 | 0.003 | 0.02 | 0.09 |
| B11 | B14 | 0 | 0 | 0 |  | B15 | B6 | 0.001 | 0.03 | 0.30 |
| B12 | B14 | 0.001 | 0.81 | 1 |  | B16 | B6 | 0.28 | 0.87 | 1 |
| B13 | B14 | 0 | 0 | 0 |  | B2 | B6 | 0.00 | 0.86 | 1 |
| B1 | B15 | 0.03 | 0.27 | 0.85 |  | B3 | B6 | 0.00 | 0.06 | 0.33 |
| B10 | B15 | 0.03 | 0.25 | 0.80 |  | B4 | B6 | 0.06 | 0.19 | 0.46 |
| B11 | B15 | 0 | 0 | 0 |  | B5 | B6 | 0.44 | 0.72 | 0.94 |
| B12 | B15 | 0.001 | 0.52 | 1 |  | B1 | B7 | 0.02 | 0.23 | 0.78 |
| B13 | B15 | <0.0001 | 0.0001 | 0.02 |  | B10 | B7 | 0.02 | 0.05 | 0.15 |
| B14 | B15 | 0.01 | 0.15 | 0.75 |  | B11 | B7 | 0.00 | 0.01 | 0.58 |
| B1 | B16 | 0.33 | 0.93 | 1 |  | B12 | B7 | 0.46 | 0.97 | 1 |
| B10 | B16 | 0.20 | 0.93 | 1 |  | B13 | B7 | 0.47 | 0.71 | 0.91 |
| B11 | B16 | 0.20 | 0.87 | 1 |  | B14 | B7 | <0.0001 | <0.0001 | 0.004 |
| B12 | B16 | 0.44 | 0.75 | 0.97 |  | B15 | B7 | <0.0001 | 0.0016 | 0.17 |
| B13 | B16 | 0.26 | 0.67 | 0.98 |  | B16 | B7 | 0.40 | 0.78 | 0.99 |
| B14 | B16 | 0.05 | 0.85 | 1 |  | B2 | B7 | 0.00 | 0.80 | 1 |
| B15 | B16 | 0.06 | 0.86 | 1 |  | B3 | B7 | 0.00 | 0.01 | 0.12 |
| B1 | B2 | 0.0005 | 0.90 | 1 |  | B4 | B7 | 0.09 | 0.44 | 0.93 |
| B10 | B2 | 0.0008 | 0.93 | 1 |  | B5 | B7 | 0.09 | 0.38 | 0.84 |
| B11 | B2 | 0 | 0 | 0 |  | B6 | B7 | 0.09 | 0.27 | 0.60 |
| B12 | B2 | <0.0001 | 0.84 | 1 |  | B1 | B8 | 0.41 | 0.68 | 0.91 |
| B13 | B2 | <0.0001 | 0.60 | 1 |  | B10 | B8 | 0.01 | 0.18 | 0.85 |
| B14 | B2 | 0.001 | 0.90 | 1 |  | B11 | B8 | <0.0001 | 0.005 | 0.12 |
| B15 | B2 | <0.0001 | 0.50 | 1 |  | B12 | B8 | 0.26 | 0.91 | 1 |
| B16 | B2 | <0.0001 | 0.87 | 1 |  | B13 | B8 | 0.27 | 0.37 | 0.47 |
| B1 | B3 | 0.36 | 0.61 | 0.86 |  | B14 | B8 | 0.02 | 0.06 | 0.17 |
| B10 | B3 | 0.49 | 0.68 | 0.86 |  | B15 | B8 | 0.01 | 0.07 | 0.33 |
| B11 | B3 | 0 | 0 | 0 |  | B16 | B8 | 0.37 | 0.92 | 1 |
| B12 | B3 | 0.01 | 0.88 | 1 |  | B2 | B8 | <0.0001 | 0.89 | 1 |
| B13 | B3 | <0.0001 | 0.002 | 0.04 |  | B3 | B8 | 0.02 | 0.15 | 0.57 |
| B14 | B3 | 0.09 | 0.52 | 0.99 |  | B4 | B8 | 0.14 | 0.33 | 0.62 |
| B15 | B3 | 0.05 | 0.36 | 0.91 |  | B5 | B8 | 0.77 | 0.92 | 0.99 |
| B16 | B3 | 0.07 | 0.92 | 1 |  | B6 | B8 | 0.33 | 0.40 | 0.48 |
| B2 | B3 | 0.001 | 0.95 | 1 |  | B7 | B8 | 0.16 | 0.25 | 0.38 |
| B1 | B4 | 0.63 | 0.81 | 0.95 |  | B1 | B9 | 0.49 | 0.76 | 0.96 |
| B10 | B4 | 0.39 | 0.86 | 1.00 |  | B10 | B9 | 0.20 | 0.48 | 0.81 |
| B11 | B4 | <0.0001 | 0.001 | 0.26 |  | B11 | B9 | <0.0001 | 0.01 | 0.13 |
| B12 | B4 | 0.11 | 0.93 | 1 |  | B12 | B9 | 0.40 | 0.92 | 1 |
| B13 | B4 | 0.13 | 0.51 | 0.95 |  | B13 | B9 | 0.48 | 0.62 | 0.75 |
| B14 | B4 | 0.08 | 0.39 | 0.88 |  | B14 | B9 | 0.04 | 0.17 | 0.47 |
| B15 | B4 | 0.04 | 0.29 | 0.81 |  | B15 | B9 | 0.02 | 0.16 | 0.59 |
| B16 | B4 | 0.28 | 0.94 | 1 |  | B16 | B9 | 0.42 | 0.88 | 1.00 |
| B2 | B4 | 0.001 | 0.92 | 1 |  | B2 | B9 | 0.0005 | 0.89 | 1 |
| B3 | B4 | 0.51 | 0.72 | 0.90 |  | B3 | B9 | 0.19 | 0.35 | 0.56 |
| B1 | B5 | 0.54 | 0.89 | 1.00 |  | B4 | B9 | 0.42 | 0.73 | 0.97 |
| B10 | B5 | 0.09 | 0.51 | 0.97 |  | B5 | B9 | 0.55 | 0.85 | 1.00 |
| B11 | B5 | 0 | 0 | 0 |  | B6 | B9 | 0.33 | 0.45 | 0.57 |
| B12 | B5 | 0.15 | 0.92 | 1 |  | B7 | B9 | 0.57 | 0.78 | 0.94 |
| B13 | B5 | 0.20 | 0.49 | 0.83 |  | B8 | B9 | 0.51 | 0.60 | 0.69 |

**Supplementary Figures**


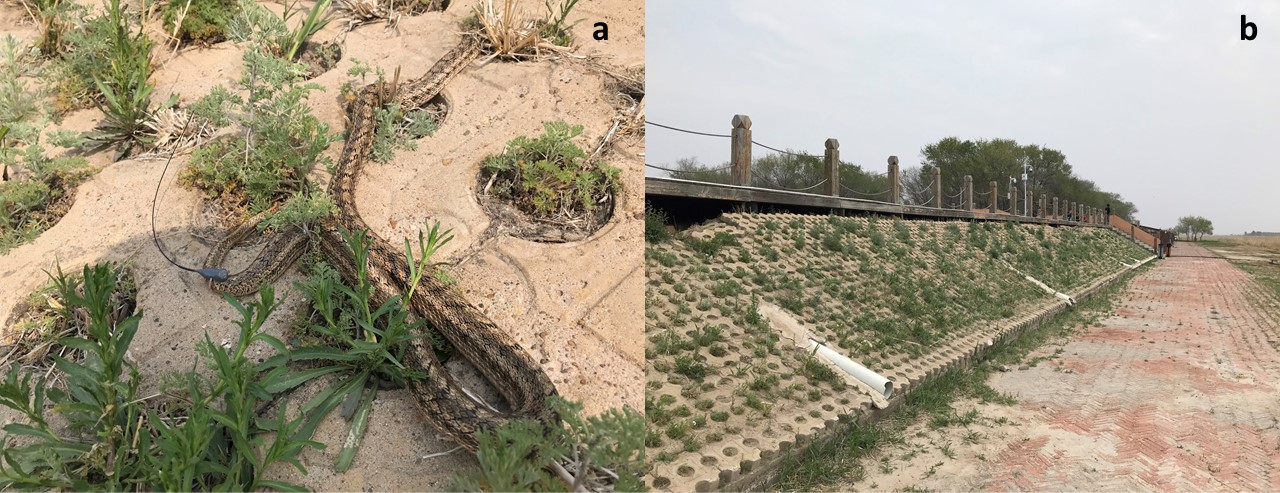


Fig. S1. — (a) A male or female? *Elaphe dione* with radio tracker attached to the base of the tail. (b) Typical aggregation habitat of *E. dione* at current study area.


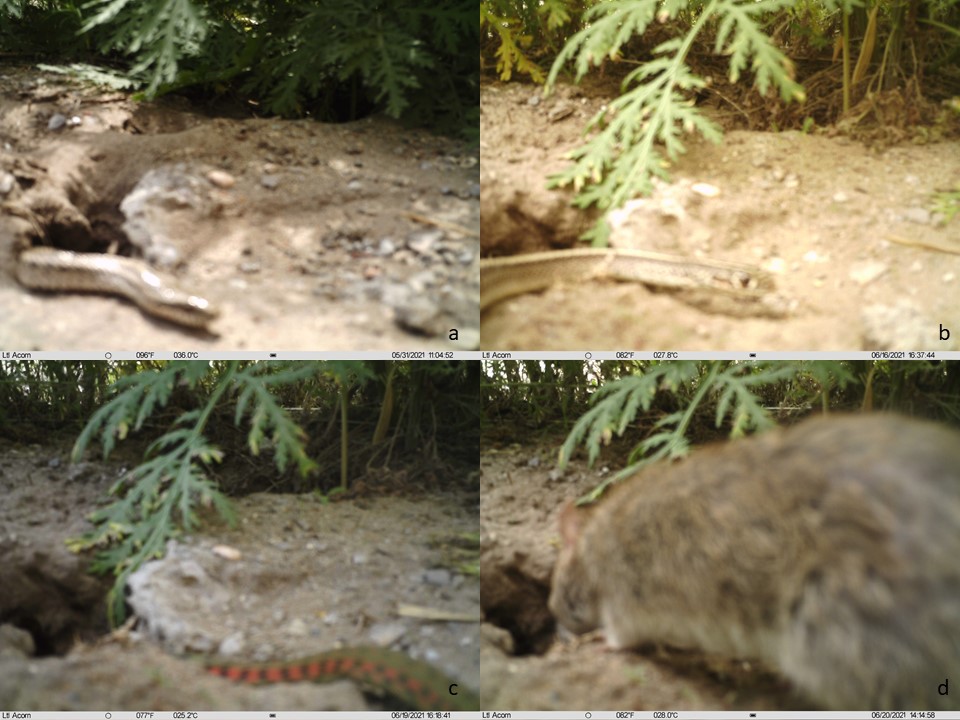


Fig. S2. — The photos of animals entering a concrete crack adjacent to a building. (a) *E. dione* (b) *Orientocoluber spinalis* (c) *Rhabdophis tigrinus* (d) *Rattus spp*


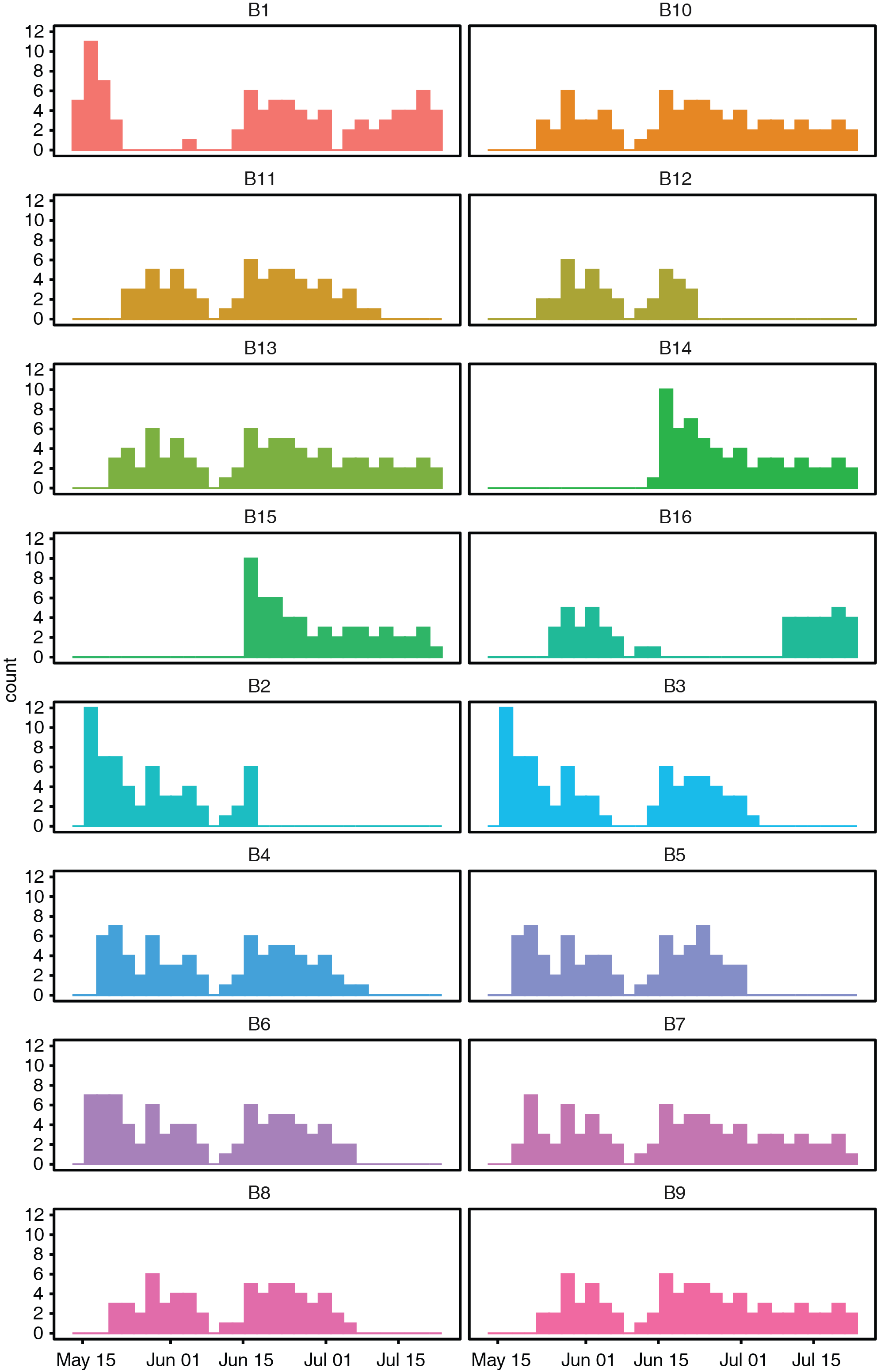


**Fig. S3.** Sampling intensity of all radio tracked adult *Elaphe dione* across the survey period (May–July).


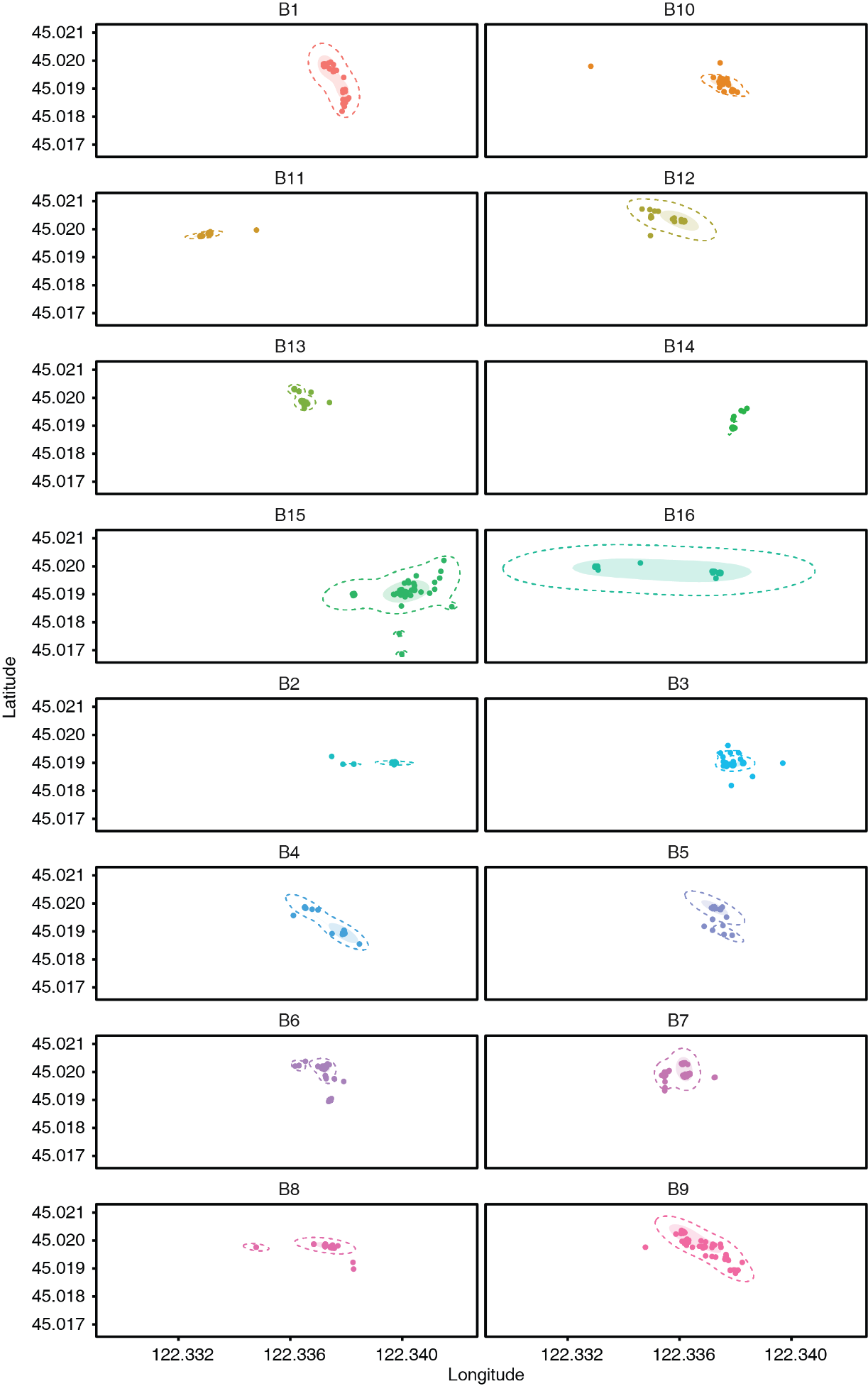


**Fig. S4.** Area-corrected, autocorrelated kernel density 95% estimates (dashed lines) of all radio tracked adult *Elaphe dione*. The 50% filled contour indicates area of core activity.
